# ATF6β is not essential for the development of physiological cardiac hypertrophy

**DOI:** 10.1371/journal.pone.0320178

**Published:** 2025-04-07

**Authors:** Emery Davis, Mohammad-Reza Shokri, Mary B. Rowland, Thomas York, Caroline T. Cohen, Anna Grace Johnson, Patrick E. Moore, Saige Schweitzer, Jon Sin, Chuong Bui, Robert N. Correll

**Affiliations:** 1 Department of Biological Sciences, University of Alabama, Tuscaloosa, Alabama, United States of America; 2 Department of Psychology, University of Alabama, Tuscaloosa, Alabama, United States of America; 3 Department of Biological Sciences, University of Texas at Dallas, Richardson, Texas, United States of America; 4 Alabama Life Research Institute, University of Alabama, Tuscaloosa, Alabama, United States of America; 5 Center for Convergent Bioscience and Medicine, University of Alabama, Tuscaloosa, Alabama, United States of America; University of Sri Jayewardenepura, SRI LANKA

## Abstract

Physiological cardiac hypertrophy is a compensatory remodeling of the heart in response to stimuli such as exercise training or pregnancy that is reversible and well-tolerated. We previously described how the activating transcription factor 6 (ATF6) proteins, ATF6α and ATF6β, were required for pathological hypertrophy in response to hemodynamic stress. Here, we examine the functional roles of both ATF6 proteins in the context of exercise-induced physiological hypertrophy. After 20 days of swim training, we found differential roles: whole body gene-deleted mice lacking ATF6α had an attenuated hypertrophic response compared to wild-type mice but those lacking ATF6β did not. Additionally, mice lacking ATF6α displayed ventricular dilation and reduced fractional shortening after swimming. While we observed no differences in the expression of downstream UPR signaling between the exercise groups, mice lacking ATF6α showed enhanced phosphorylation of extracellular signal-regulated kinase 1/2 (ERK1/2). Thus, in response to swim training, loss of ATF6β did not hinder the development of physiological hypertrophy, but loss of ATF6α resulted in significantly reduced cardiac fractional shortening.

## Introduction

Cardiac hypertrophy is characterized by an increase in the size of individual cardiomyocytes and the overall muscle mass of the heart. This develops as a compensatory response to maintain cardiac output during stress conditions [1]. Physiological hypertrophy, which is observed during pregnancy or exercise, is reversible and well-tolerated [2]. It often involves signaling through the PI3K/Akt and RHEB/mTORC1 pathways [3,4]. Conversely, pathological hypertrophy results from disease stimuli such as prolonged hypertension [5] and is commonly regulated through the calcineurin/NFAT and CaMKII/MEF-2 pathways [5,6]. While the molecular pathways controlling physiological and pathological hypertrophy are generally distinct, there are signaling molecules, such as GSK-3 and PIP_2_, that participate in and control both processes [3]. Thus, some important regulators of pathological hypertrophy could also be involved in regulating physiological hypertrophy.

It was recently identified that the activating transcription factor 6 (ATF6) pathway is involved in the cardiac hypertrophic response [7–9]. The ATF6 proteins consist of ATF6α and ATF6β, encoded by the *Atf6* and *Atf6b* genes, respectively [10]. ATF6 is one of the three main arms of the UPR, along with inositol-requiring enzyme 1α (IRE1α) and protein kinase RNA-activated-like ER kinase (PERK) [11]. Each player acts as a unique ER-stress sensor, resulting in distinct downstream effects [11]. Perturbations to ER homeostasis lead to the accumulation of misfolded proteins, and this impaired proteostasis activates the ATF6 arm of the UPR. ATF6 is then translocated from the ER membrane to the Golgi, where the protein is cleaved, and the resulting transcriptionally active N-terminal fragment is trafficked to the nucleus to regulate gene expression [10]. ATF6α is a robust but short-lived transcription factor due to its VN8 domain and is widely synonymous with “ATF6 signaling”. ATF6β, which lacks the VN8 domain, is a longer-lived but less potent transcription factor [12] and because of its lower transcriptional activity, was suggested to be dispensable for UPR signaling or even serve as an inhibitor of ATF6α in the heart [13–15]. However, recent studies found that both ATF6 paralogs are required for pathological hypertrophy and control partially overlapping gene targets, including ER protein chaperones and ER-associated degradation proteins [8,16]. Gene-deleted mice lacking *Atf6* or *Atf6b* showed a significant reduction in cardiac hypertrophy two weeks after transverse aortic constriction (TAC) and experienced more rapid decompensation and heart failure after eight weeks of TAC [8]. Loss of ATF6α or ATF6β also resulted in reduced activation of ATF6 targets such as the ER chaperone calreticulin [8]. Furthermore, another study found that *Atf6*-deleted mice had a reduced ability to develop physiological hypertrophy induced by free wheel running and that ATF6α regulated cardiac hypertrophy via non-canonical Rheb/mTORC1 signaling. However, the role of ATF6β was not examined [7].

Here, we demonstrate differential roles of ATF6α and ATF6β in regard to physiological hypertrophy. Deletion of *Atf6b* did not inhibit the development of physiological hypertrophy elicited by swimming exercise compared to wild-type (Wt) controls. However, *Atf6*-null mice were unable to fully develop hypertrophy after exercise and also exhibited left ventricle chamber dilation and reduced cardiac function, similar to the early stages of decompensation. While we detected no significant changes in the expression of ER protein chaperones at the 20-day timepoint, we found that ERK1/2 phosphorylation was increased in the hearts of *Atf6*-null mice, perhaps as a compensatory mechanism. Thus, these data demonstrate that ATF6β is not required for the development of physiological hypertrophy and that loss of ATF6α may shift the heart towards pathological remodeling after exercise training.

## Materials and methods

### Gene-deleted mice

Whole body gene-deleted mice lacking *Atf6* (*Atf6*^-/-^) or *Atf6b* (*Atf6b*^-/-^) were generated in the laboratory of Kazutoshi Mori (Kyoto University, Kyoto, Japan) [17]. All mice used were between 3 and 6 months of age and in the C57BL/6 background. Wt littermate mice served as controls. Both male and female mice were used in the study, and all animals were included in the statistical analyses. For some experiments, sedentary mice (termed the Rest group) of the same genotypes served as controls. All animal experiments were approved by the University of Alabama Institutional Animal Care and Use Committee and were in accordance with the National Institutes of Health guidelines (Protocol # 18-08-1483 and 18-08-1487).

### Swimming protocol

**Fig 1:**
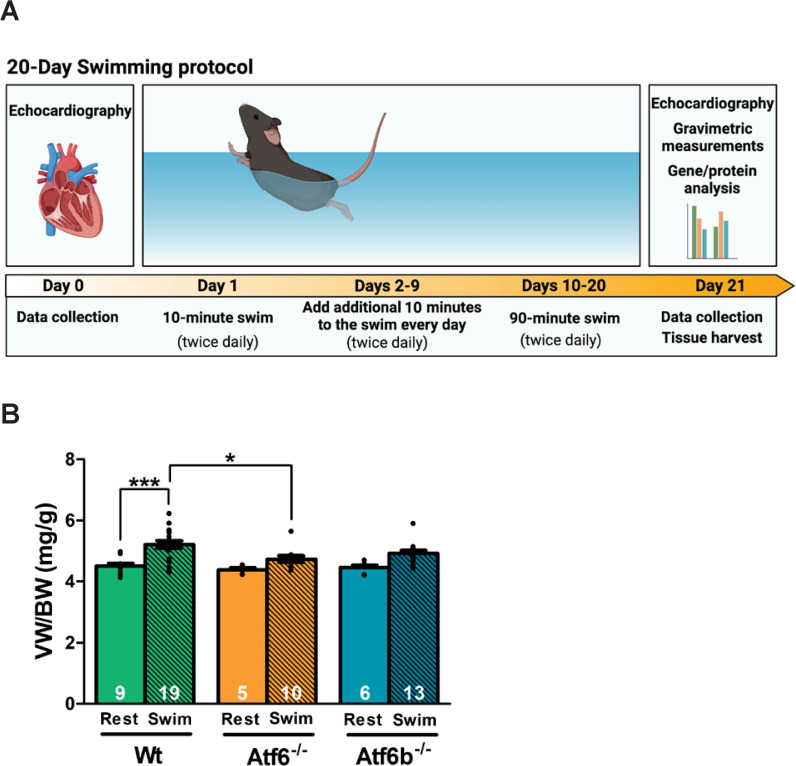
Swim training resulted in increases in ventricle weight. (A) Diagram depicting swim training protocol and timing of echocardiography and heart tissue harvest. (B) Gravimetric measurement of ventricle weight normalized to body weight (VW/BW). Number of mice used is shown in the graph. Rest represents non-exercised mice and Swim represents post-exercise mice. * p <  0.05; ***p <  0.001.

Day 1 of the swimming protocol encompassed two 10-minute sessions in pre-warmed water, separated by a minimum four-hour interval. The duration of swimming sessions was extended by 10 minutes each day through day 9, when the mice participated in two 90-minute swimming sessions. On days 10-20, the duration remained at 90 minutes per session, twice a day, as previously described [18], and on day 21 subsequent experiments were performed (Fig 1A). 4 male mice died during the swimming protocol (2 *Atf6*^-/-^ and 2 *Atf6b*^-/-^). Sedentary (Rest) mice and swim-trained mice on day 21 were euthanized via CO_2_ inhalation, with cervical dislocation as a secondary method, in accordance with the National Institutes of Health guidelines (Protocol # 18-08-1483 and 18-08-1487). Gravimetric data was collected consisting of body weight, total heart weight, ventricle weight, and lung weight. Following the excision of the heart, the superior and inferior portions of the ventricles were carefully separated using a scalpel. The superior portion of the ventricles was fixed in 10% formalin and used for histology studies, while the inferior portion was promptly frozen in liquid nitrogen and stored at -80°C for immunoblotting studies and gene expression analyses. 3 samples from each group were used for immunoblotting, and the rest were used for gene-expression analysis. Samples chosen for these assessments were randomized within each genotype.

### Echocardiography

For echocardiography procedures, only swim-trained mice were assessed. Echocardiography was performed on all mice on day 0 before the start of the swimming protocol and again on day 21, within 24 hours after protocol completion. The assessments were conducted in a blinded manner (Fig 1A). The mice were initially anesthetized in an inhalation chamber with 2% isoflurane combined with 1.5 L/min of oxygen. To maintain anesthesia, the mice were placed on a heating pad and connected to the anesthesia breathing system via nose cone. Heart function and structure were evaluated through parasternal short axis M-mode echocardiography using a Philips iU22 ultrasound system (Philips Healthcare, Andover, MA, USA) with L15-7io compact linear array transducer (Philips, 15-7 MHz). Ventricular measurements were taken in M-mode across three distinct cardiac cycles, and the resulting values were averaged.

### Western blotting

For immunoblotting procedures, only swim-trained mice were assessed. Immunoblotting was performed using heart lysates composed of the inferior region of the left and right ventricle. Ventricles were homogenized using a Tissue Master 125 (Omni International, Kennesaw, GA, USA) in buffer consisting of 20 mM Tris-HCl (pH 7.5), 250 mM NaCl, 1% Triton X-100, 0.5 mM dithiothreitol (DTT), and HALT 1x protease inhibitor cocktail [8]. Samples were sonicated and centrifuged at 21,000 RCF for 10 minutes at 4°C. Resulting supernatants were transferred to new tubes and their protein concentrations were quantified using the Bradford assay (Sigma-Aldrich, St. Louis, MO, USA). Subsequently, 10-50 µg of each sample were subjected to SDS-PAGE and transferred to PVDF membranes. Membranes were subjected to immunoblotting with primary antibodies followed by HRP-conjugated secondary antibodies. The blots were imaged using Radiance ECL (Azure Biosystems, Dublin, CA, USA) on an Azure C300 imaging system (Azure Biosystems) with the chemiluminescence setting. The primary antibodies used were: GAPDH (10R-G109a), 1:20,000 (Fitzgerald Industries International INC., Acton, MA, USA); GRP78/BiP (ET-21), 1:1000 (Sigma-Aldrich); GRP94/HSP90B1, 1:1000 (Sigma-Aldrich); calreticulin, 1:1000 (Cell Signaling Technology, Danvers, MA, USA); p44/42 MAPK (Erk1/2), 1:1,000 (Cell Signaling Technology); P-p44/42 MAPK (T202/Y204), 1:1,000 (Cell Signaling Technology); Akt (pan) (C67E7), 1:1,000 (Cell Signaling Technology); P-Akt (S473) (D9E) XP(R), 1:1,000 (Cell Signaling Technology); AceCS1 (D19C6) #3658, 1:1,000 (Cell Signaling Technology); Phospho-Acetyl-CoA Carboxylase (Ser79) (D7D11) #11818, 1:1000 (Cell Signaling Technology); Acetyl-CoA Carboxylase (C83B10) #3676, 1:1000 (Cell Signaling Technology); ATP-Citrate Lyase #4332, 1:1000 (Cell Signaling Technology); Phospho-ATP-Citrate Lyase (Ser455) #4331, 1:1000 (Cell Signaling Technology); Fatty Acid Synthase (C20G5) #3180, 1:1000 (Cell Signaling Technology); Lipin 1 (D2W9G) #14906, 1:1000 (Cell Signaling Technology); ACSL1 (D2H5) #9189, 1:1000 (Cell Signaling Technology); PKM2 (D78A4) XP® #4053, 1:1000 (Cell Signaling Technology); Pyruvate Dehydrogenase (C54G1) #3205, 1:1000 (Cell Signaling Technology); Hexokinase I (C35C4) #2024, 1:1000 (Cell Signaling Technology); Hexokinase II (C64G5) #2867, 1:1000 (Cell Signaling Technology); LDHA (C4B5) #3582, 1:1000 (Cell Signaling Technology); PKM1/2 (C103A3) #3190, 1:1000 (Cell Signaling Technology); and PFKP (D4B2) #8164, 1:1000 (Cell Signaling Technology). Secondary antibodies used were: HRP Secondary Antibody Goat anti-Rabbit IgG, 1:5,000 (Azure Biosystems) and HRP Secondary Antibody Goat anti-Mouse IgG, 1:5,000 (Azure Biosystems). Uncropped blots are available in S2–S4 Figs.

### Gene expression analysis

For gene expression analyses, both sedentary (Rest) and swim-trained mice were assessed. Heart tissue composed of the inferior region of the right and left ventricle were homogenized with a FastPrep-24 instrument (MP Biomedicals, Santa Ana, USA) using 3 stainless steel beads per sample for 30 s at 6 m/s. Total RNA was extracted from mouse heart ventricles using RNeasy Fibrous Tissue Mini Kit (Qiagen, Hilden, Germany), according to the manufacturer’s instructions. Subsequently, cDNA synthesis was performed with 1 µg of RNA from each sample using SuperScript IV VILO Master Mix with ezDNase Enzyme (Thermo Fisher Scientific, Waltham, MA, USA), according to the manufacturer’s protocol. For quantitative polymerase chain reaction (qPCR), the SYBR Green qPCR 2X Master Mix (Intact Genomics, St. Louis, MO, USA) was used and amplification was completed on the CFX96 real-time System (Bio-Rad). Previously described primers used were *Manf* [19], *Calr* [20], *Grp78* [21], *Nppa* [22], *Nppb* [22], *Actc1* [23], *Myh6* [24], *Myh7* [25], *Igfr1* [26], *Hgf* [26], *Gata4* [27], *Tnni3* [27], *Cited4* [28], *Cebpb* [27], *Hand2* [29], and *Rheb* [30]. The complete list of qRT-PCR primers used in this study are listed in Table 1. Relative gene expression was calculated using 2 ^–(ΔΔCT)^ method and *Gapdh* served as the reference gene for all samples.

**Table 1:** qPCR primer sequences.

Gene	Sequence	
	Forward	Reverse
*Manf*	GACAGCCAGATCTGTGAACTAAAA	TTTCACCCGGAGCTTCTTC
*Calr*	GAATACAAGGGCGAGTGGAA	GGGGGAGTATTCAGGGTTGT
*Hspa5*	CACGTCCAACCCCGAGAA	ATTCCAAGTGCGTCCGATG
*Hsp90b1*	GGGAGGTCACCTTCAAGTCG	CTCGAGGTGCAGATGTGGG
*Adgrg3*	CACCTTCGACTTGAATGACACTGCTC	TGCTGATGTTCTGGATCAATGCCTT
*Sdf2l1*	CTATCCAACAACCAGGAGGTGAG	GTTCACCAGTGACCGACAGG
*Nppa*	TTCCTCGTCTTGGCCTTTTG	CCTCATCTTCTACCGGCATC
*Nppb*	GTCCAGCAGAGACCTCAAAA	AGGCAGAGTCAGAAACTGGA
*Actc1*	AAGATCAAGATTATTGCTCCCCCTG	GGTCATCCTGAATATAAGGTAGGCT
*Myh6*	CAATGCAGAGTCGGTGAAGG	CCTCTGTCTGGTAGGTGAGC
*Myh7*	ACTGTCAACACTAAGAGGGTCA	TTGGATGATTTGATCTTCCAGGG
*Igf1r*	GTGGGGGCTCGTGTTTCTC	GATCACCGTGCAGTTTTCCA
*Hgf*	ATGTGGGGGACCAAACTTCTG	GGATGGCGACATGAAGCAG
*Gata4*	GGGCCCTCTTTGTCATTCTTC	TCCTTGCTTTCTGCCTGCTAC
*Tnni3*	TCTATGACCTCCGTGGCAAGT	TCCTCCTTCTTCACCTGCTTG
*Cited4*	CATGGACACCGAGCTCATC	CTGACCCCAGGTCTGAGAAG
*Cebpb*	GGGGTTGTTGATGTTTTTGGT	TCGAAACGGAAAAGGTTCTCA
*Hand2*	CCGACACCAAACTCTCCAAG	TCTTGTCGTTGCTGCTCACT
*Rheb*	CGGTCTGTGGGAAAGTCCTC	ATATTCATCCTGCCCCGCTG
*Gapdh*	GGTGTGAACGGATTTGGCC	TGAAGGGGTCGTTGATGGC

### Histology

Trichrome staining was performed on deparaffinized heart sections, composed of the superior portion of the right and left ventricle, using Trichrome Stain (Masson) Kit (#HT15, Sigma-Aldrich) according to the manufacturer’s protocol. Images were captured using a Nikon Ti2 inverted epifluorescence microscope equipped with a Qi2 camera (Nikon, Tokyo, Japan).

### Statistics

Regression analysis was used to compare mice of different genotypes after 20 days of swimming in terms of echocardiographic measurements (e.g., intraventricular septum thickness, ventricular posterior wall thickness). Particularly, beta regression with a logit link was used to examine if different genotypes exhibited differences in fractional shortening (FS). Beta regression was chosen because fractional shortening (FS) was bounded by 0 and 1. For all other echocardiographic measurements, linear regression with robust standard errors was used. In each regression analysis, the dependent variable was the measurement taken on day 20 (e.g., intraventricular septum thickness taken on day 20). The independent variable of interest was genotypes. As genotypes had 3 categories (i.e., Wt, *Atf6*^-/-^ and *Atf6b*^-/-^), there were 3 comparisons (i.e., *Atf6*^-/-^ vs. Wt; *Atf6b*^-/-^ vs. Wt; and *Atf6*^-/-^ vs. *Atf6b*^-/-^). The baseline measurement (taken on day 0) was included as a covariate. The analyses were carried out using PROC GLIMMIX in SAS/STAT 15.1. All other statistical analyses were performed using GraphPrism 5 software (Graphpad Software, La Jolla, CA, USA). The values are presented as mean ±  SEM. The multiple group comparisons were performed using a one-way ANOVA coupled with Newman-Keuls multiple comparisons test. Level of significance was 5%.

## Results

**Fig 2:**
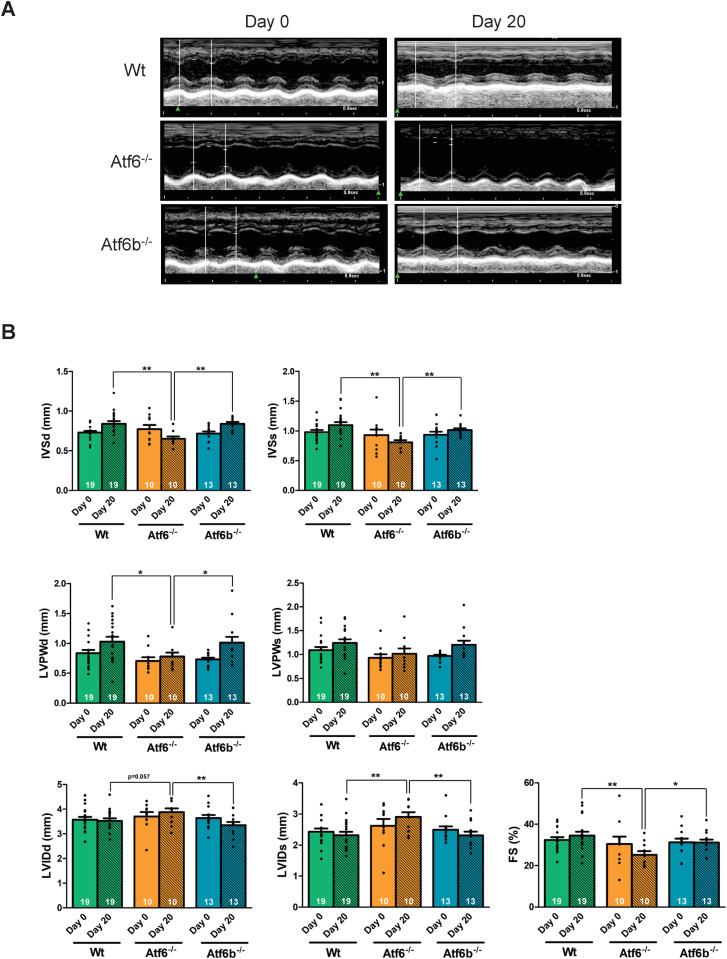
Swim training resulted in maladaptive cardiac remodeling in *Atf6*^*-/-*^ mice. (A) Representative echocardiographic images and visualization of echocardiographic measurements presented in Table 2 showing (B) left ventricular posterior wall thickness in diastole (LVPWd) and systole (LVPWs), (C) intraventricular septum in diastole (IVSd) and systole (IVSs), (D) left ventricular internal dimension in diastole (LVIDd) and systole (LVIDs), and (E) fractional shortening percentage (FS%). The number of mice analyzed is given within the graph. Day 0 is the pre-exercise measurement, and day 20 is the post-exercise measurement. Regression analysis was used to compare echocardiographic parameters between genotypes after 20 days of swimming (Table 2). * p <  0.05; **p <  0.01.

To determine if ATF6α and ATF6β are required for physiological hypertrophy, we enrolled whole body gene-deleted mice lacking *Atf6* (N = 10), *Atf6b* (N = 13), and Wt controls (N = 19) into a 20-day swimming protocol (Fig 1A) [18]. Echocardiography was performed on days 0 and 21, followed by tissue harvest and gravimetric measurements. Physiological hypertrophy was assessed by an increase in ventricle weight. Wt mice demonstrated a significant increase in ventricle weight to body weight (VW/BW) ratio after swim training compared to rest mice, the *Atf6*^-/-^ mice showed a significantly attenuated VW/BW ratio after swim training compared to Wt swim mice, but the *Atf6b*^-/-^ mice demonstrated only a trend towards increased hypertrophy (Fig 1B). Table 2 presents results from regression analyses that compared different genotypes after 20 days of swimming in terms of echocardiographic measurements. Fig 2 accompanies Table 2 to provide a graphical presentation of how genotypes differ. Regression analyses revealed that swimming-induced physiological cardiac remodeling was significantly different in the *Atf6*^-/-^ mice compared to Wt and *Atf6b*^-/-^ mice (Fig 2 and Table 2). Specifically, *Atf6*^-/-^ mice showed a significant reduction in intraventricular septum (IVS) thickness in both systole and diastole, significantly reduced left ventricular posterior wall (LVPW) thickness in diastole, a significant increase in left ventricular internal dimension (LVID) in systole, and a significant reduction in cardiac function as measured by fractional shortening (FS), compared to Wt and *Atf6b*^-/-^ groups. Additionally, *Atf6*^-/-^ mice showed a significant increase in LVID in diastole compared to *Atf6b*^-/-^ mice and a strong trend towards increased LVID in diastole compared to Wt mice (Fig 2 and Table 2). Because of the significant reduction in fractional shortening, and significant increase in LVID in the *Atf6*^-/-^ mice compared to Wt and *Atf6b*^-/-^ mice, trichrome staining was completed to evaluate the presence of fibrosis. However, minimal fibrosis was found in all groups after swim training (S1 Fig). Taken together, these data suggest that swim training does not induce the expected physiological hypertrophic remodeling in *Atf6*^-/-^ mice. Indeed, *Atf6*^-/-^ mice not only fail to show hypertrophic remodeling after exercise, but also have left ventricle chamber dilation, as measured by LVID, and reduced fractional shortening that is commonly associated with cardiac decompensation.

**Table 2:** Regression analysis comparing echocardiographic parameters between genotypes after 20 days of swimming, controlling for day 0 measurement, Wt was used as the reference category. This analysis was used to determine statistical significance showed in Fig 2. b = regression coefficient, se = standard error, p = p-value. * p <  0.05, **p <  0.01.

	IVSd			LVPWd			LVIDd		
	b	se	p	b	se	p	b	se	p
Day 0 measurement	-0.024	0.151	0.877	0.152	0.327	0.646	0.12	0.14	0.398
Atf6^-/-^ vs. Wt	-0.188	0.043	**<.01**	-0.23	0.112	**0.047**	0.337	0.172	0.057
Atf6b^-/-^ vs. Wt	-0.002	0.041	0.966	-0.002	0.12	0.989	-0.181	0.159	0.262
Atf6^-/-^ vs. Atf6b^-/-^	-0.186	0.039	**<.01**	-0.228	0.111	**0.048**	0.518	0.189	**<.01**
	**IVSs**			**LVPWs**			**LVIDs**		
	b	se	p	b	se	p	b	se	p
Day 0 measurement	-0.045	0.103	0.666	0.364	0.233	0.126	0.054	0.157	0.731
Atf6^-/-^ vs. Wt	-0.292	0.059	**<.01**	-0.169	0.126	0.188	0.58	0.181	**<.01**
Atf6b^-/-^ vs. Wt	-0.086	0.057	0.142	0.004	0.114	0.971	-0.006	0.154	0.972
Atf6^-/-^ vs. Atf6b^-/-^	-0.206	0.043	**<.01**	-0.173	0.123	0.167	0.586	0.189	**<.01**
	**FS**								
	b	se	p						
Day 0 measurement	0.081	0.658	0.903						
Atf6^-/-^ vs. Wt	-0.436	0.121	**<.01**						
Atf6b^-/-^ vs. Wt	-0.14	0.106	0.197						
Atf6^-/-^ vs. Atf6b^-/-^	-0.296	0.13	**0.028**						

**Fig 3:**
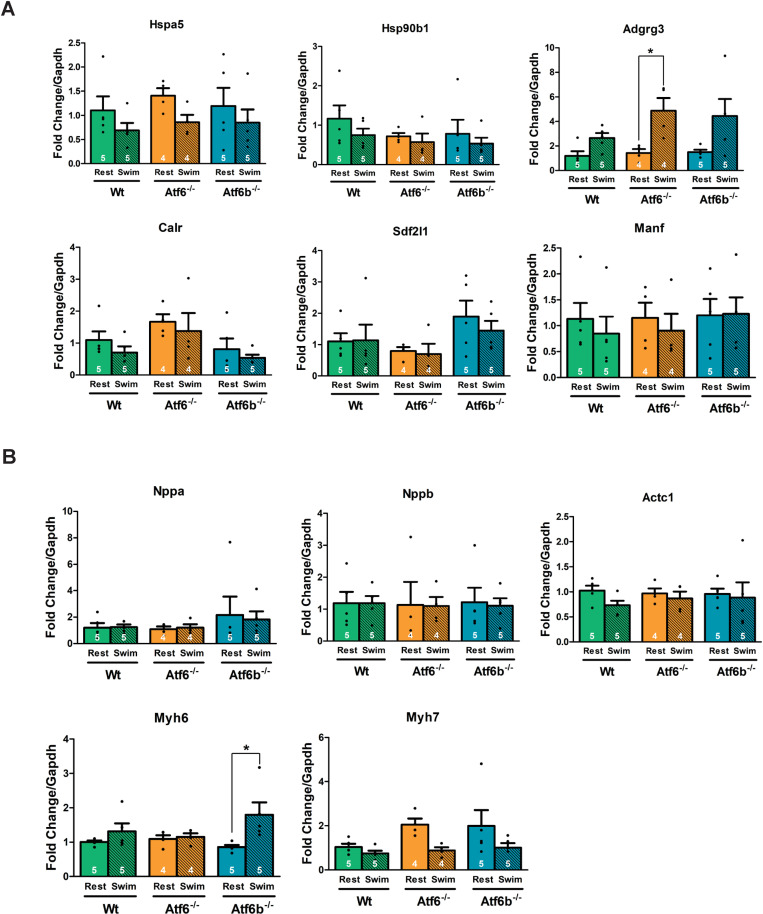
Expression of UPR- and inflammation-associated genes and genes associated with pathological hypertrophy following swim training. qPCR was used to assess (A) the expression of ER stress- and inflammation-associated genes and (B) the pathological hypertrophy-associated genes in sedentary (rest) and swim-trained Wt, *Atf6*^-/-^, and *Atf6b*^-/-^ ventricles. *Gapdh* served as the reference gene for all samples. Number of mice used is shown in the graph. Rest represents non-exercised mice and Swim represents post-exercise mice. * p <  0.05.

**Fig 4:**
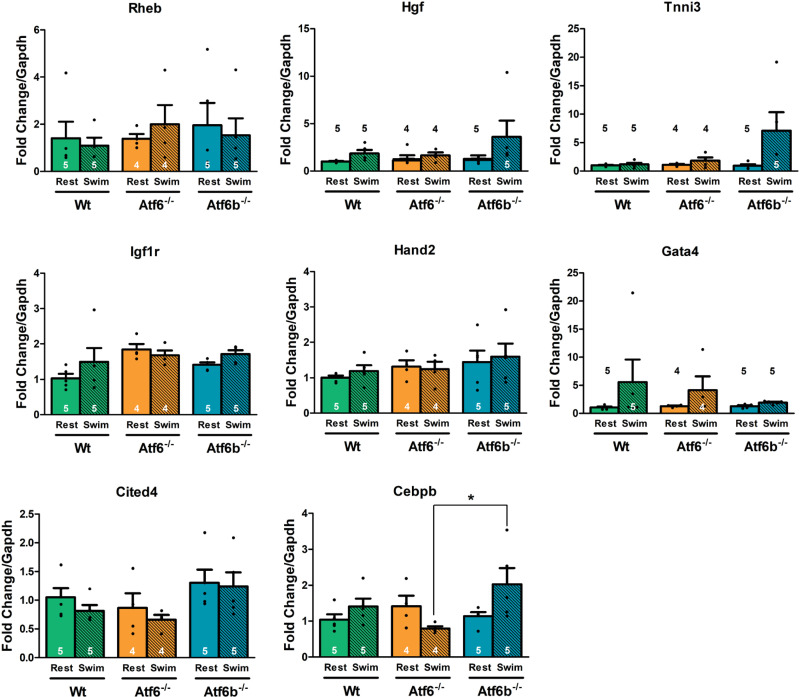
Expression of genes associated with physiological hypertrophy after swim training. qPCR was used to assess the expression of genes associated with physiological hypertrophy in sedentary and swim-trained Wt, *Atf6*^-/-^, and *Atf6b*^-/-^ ventricles. *Gapdh* served as the reference gene for all samples. Number of mice used is shown in the graph. Rest represents non-exercised mice and Swim represents post-exercise mice. * p <  0.05.

To determine if there were any differences in UPR- or inflammation-associated gene expression between groups due to swim training, RNA was extracted from the ventricles of rested and swim-trained Wt, *Atf6*^-/-^, and *Atf6b*^-/-^ mice to examine gene expression via qPCR. A significant difference was only detected in the expression of *Adgrg3* (expressing GPR97), which was upregulated in exercised *Atf6*^-/-^ mice but was not significantly different in Wt or *Atf6b*^-/-^ mice following swim training (Fig 3A). Because exercised *Atf6*^-/-^ mice showed features of maladaptive remodeling, expression levels of fetal gene program markers were evaluated due to their association with pathological cardiac hypertrophy [6]. *Myh6* expression was significantly upregulated in exercised *Atf6b*^*-/-*^ mice, but it was not significantly different in Wt or *Atf6*^*-/-*^ mice after swim training. (Fig 3B). We used qPCR to determine if there was differential expression of genes associated with physiological cardiac hypertrophy such as *Rheb* [7], *Hgf* [26], *Tnni3* [27], *Igf1r* [26], *Hand2* [31], *Gata4* [27], *Cited4* [28], and *Cebpb* [27] in mice following swim training. We detected a significant difference in the expression of *Cebpb* between *Atf6*^-/-^ and *Atf6b*^-/-^ mice after swim training (Fig 4).

**Fig 5:**
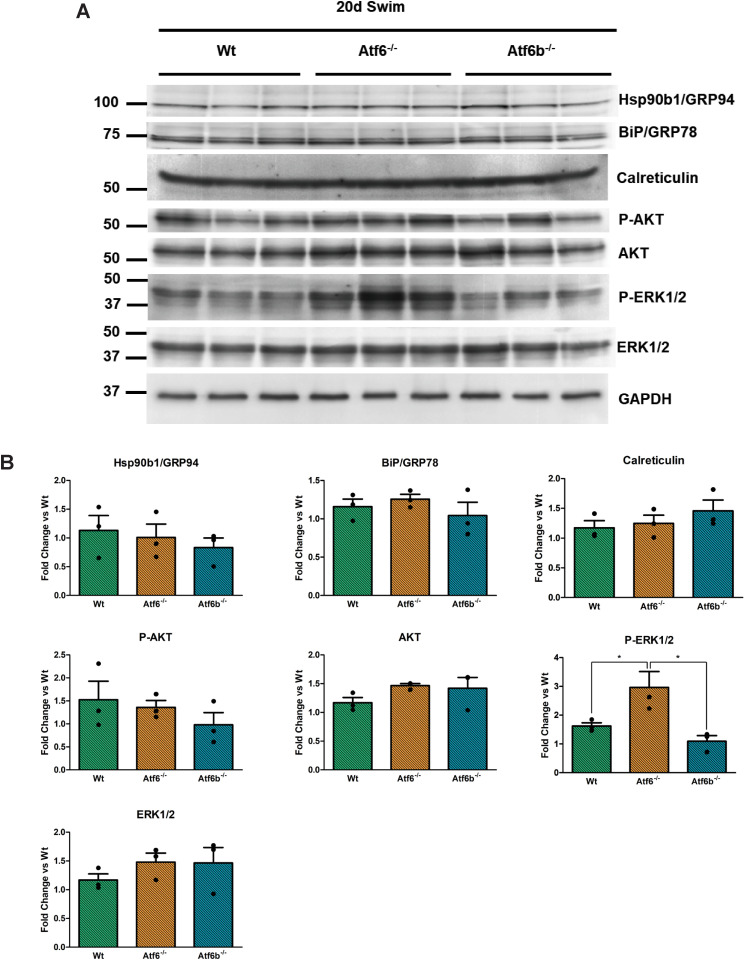
Swim training resulted in enhanced ERK1/2 phosphorylation in *Atf6*^-/-^ mice. Hearts from Wt, *Atf6*^-/-^, and *Atf6b*^-/-^ were harvested following 20 days of swim training and (A) immunoblotted for ER stress-associated ATF6 targets and proteins associated with hypertrophic growth of the heart. (B) Quantification was performed using AzureSpot Pro software, with GAPDH serving as the reference protein. p-AKT and p-ERK1/2 are plotted relative to total AKT and ERK1/2, respectively. Molecular weight markers (in kD) shown to the left of the blots. * p <  0.05.

**Fig 6:**
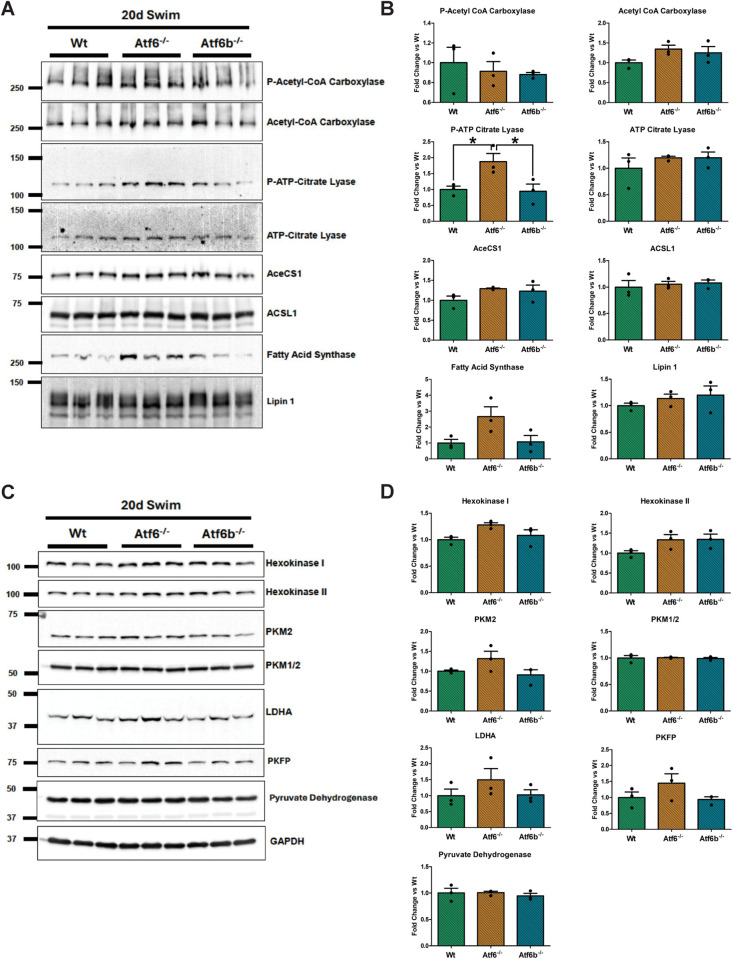
The effect of swim training on expression of markers of fatty acid oxidation and glycolysis. (A) Immunoblots of fatty acid oxidation markers, (B) quantification of fatty acid oxidation blots, (C) immunoblots of glycolysis markers, (D) quantification of glycolysis blots. Quantification was performed using AzureSpot Pro software, with GAPDH serving as the reference protein. p-Acetyl-CoA Carboxylase and p-ATP-Citrate Lyase are plotted relative to total Acetyl-CoA Carboxylase and ATP-Citrate Lyase, respectively. Molecular weight markers (in kD) shown to the left of the blots. * p <  0.05.

Western blotting confirmed that the protein expression of ER stress-associated ATF6 targets, including GRP78/BiP, Hsp90b1/GRP94, and calreticulin [8] were not differentially expressed between the genotypes after 20 days of swim training (Fig 5). AKT and P-AKT, which are required for physiological hypertrophy [32], were also not different between the genotypes after swim training (Fig 5). However, phosphorylation of ERK1/2 was increased in the exercised *Atf6*^-/-^ mice compared to exercised Wt and *Atf6b*^-/-^ mice (Fig 5B). To see if exercise resulted in changes in cardiac metabolism, we immunoblotted for markers of fatty acid oxidation and glycolysis and found phosphorylation of ATP-citrate lyase (ACLY) was significantly upregulated in the *Atf6*^-/-^ mice compared to the Wt and *Atf6b*^-/-^ mice (Fig 6).

## Discussion

Both ischemic and hemodynamic stress are associated with cardiac remodeling and ER stress, resulting in activation of the UPR through its three major signaling pathways: IRE1, PERK, and ATF6 [33]. In recent years, multiple studies have identified ATF6 signaling as being broadly protective in the context of cardiac disease. Activation of ATF6 via transgenic expression of an activated form [21], overexpression of its obligate regulator thrombospondin 4 (Thbs4) [9], or using pharmacological means [34] has been shown to improve survival and cardiac function after ischemic injury. It has also been identified that ATF6 signaling is required for pathological cardiac hypertrophy [7,8]. Mice with global deletion of *Atf6* or *Atf6b* exhibited reduced expression of ER protein chaperones following TAC, which led to rapid decompensation and subsequent heart failure [8]. This was attributed to reduced ability to maintain cardiac myocyte proteostasis under hemodynamic stress. Additionally, it was demonstrated that hemodynamic stress resulted in ATF6α-dependent upregulation of the mTOR pathway signaling protein Rheb [7]. Conditional deletion of *Atf6* in cardiomyocytes prevented this increase in Rheb expression, inhibiting mTORC1 activity during a hypertrophic growth stimulus and prevented cardiac remodeling [7]. This was also true for physiological hypertrophy, where cardiac-specific deletion of *Atf6* prevented exercise-induced remodeling using a voluntary free wheel-running protocol [7]. However, a potential role for ATF6β in physiological hypertrophy was not examined in that study. Given that we previously demonstrated ATF6β has similar importance to ATF6⍺ in the regulation of pathological hypertrophy [8], we sought to determine whether both ATF6 proteins are also required for the development of physiological hypertrophy.

To explore this, we employed a 20-day swimming protocol. This protocol has been previously demonstrated to reliably elicit exercise-induced remodeling and ensures all genotypes exercise for the same period of time [18]. We found that after exercise, echocardiographic changes associated with physiological hypertrophy in *Atf6b*^-/-^ mice were not significantly different from those changes in Wt mice. Thus, ATF6β does not appear to be required for the development of physiological hypertrophy, even though it has been shown to be required for the development of pathological hypertrophy [8]. That said, while the echocardiographic data showed no significant differences between *Atf6b*^-/-^ and WT mice, mice lacking *Atf6b* did not show a significantly increased VW/BW after swimming whereas Wt mice did show statistically significant hypertrophy (Fig 1B). Additionally, exercised *Atf6b*^*-/-*^ mice showed significantly increased *Myh6* expression compared to rested *Atf6b*^-/-^ mice with no significant changes in *Myh7* expression (Fig 3). This is unsurprising, as an increase in *Myh6* expression was previously identified after swim training [27] and the echocardiography data supports a normal physiological hypertrophy profile for *Atf6b*^*-/-*^ mice, which would not be associated with switch from α-MHC to β-MHC expression [35].

Conversely, echocardiography measurements from *Atf6*^-/-^ mice showed a significantly different response to swim training compared to Wt and *Atf6b*^-/-^ mice, suggesting an inability to develop physiological hypertrophy, and supports previous work demonstrating that ATF6α is required for the development of physiological hypertrophy stimulated by free wheel-running exercise [7]. Additionally, *Atf6*^-/-^ mice showed a significant increase in LVIDd compared to *Atf6b*^*-/-*^ mice, a significant increase in LVIDs compared to Wt and *Atf6b*^*-/-*^ mice, and a significant reduction in fractional shortening compared to Wt and *Atf6b*^*-/-*^ mice. This is intriguing, as the echocardiographic parameters observed in *Atf6*^-/-^ mice are not just indicative of compromised physiological hypertrophy, but actually resemble cardiac decompensation. However, the presence of minimal fibrosis (S1 Fig) indicates this may represent an early stage of decompensation because fibrotic scarring is associated with late-stage decompensation and heart failure. Mice lacking *Atf6* also demonstrated changes in the expression of some genes, including a significant decrease in *Cebpb* expression (Fig 4) compared to *Atf6b*^-/-^ mice after swim training. A decrease in *Cebpb* expression was previously associated with physiological hypertrophy [27], and in fact *Cebpb* has been suggested as a “master regulator” of physiological hypertrophy [36]. Additionally, decreased expression of *Cebpb* has been shown to be protective against pathological remodeling [27,37]. However, these effects are counter to the echocardiographic changes we observed in *Atf6*^-/-^ mice after swim training. Additionally, *Adgrg3* (encoding GPR97) was significantly upregulated in *Atf6*^-/-^ mice after swim-training (Fig 3). While GPR97 is highly expressed in the heart [38,39] and has been implicated in pro-inflammatory responses in leukocytes [40–42], its functions are not fully understood.

At the protein level, ERK1/2 phosphorylation expression was significantly upregulated in the swim-trained *Atf6*^-/-^ mice compared to the *Atf6b*^-/-^ and Wt mice (Fig 5B). ERK1/2 has a robust scientific literature and has been extensively examined in the context of cardiac hypertrophy, where it appears to control the geometry of the hypertrophic response [43,44]. Activation of ERK1/2 signaling via MEK1 overexpression resulted in increased cardiomyocyte thickness and promoted concentric cardiac hypertrophy, but gene-deleted mice lacking ERK1/2 displayed long and thin myocytes, promoting eccentric hypertrophy [43]. While ERK1/2 has been shown to be dispensable for swim-induced cardiac hypertrophy [45], and some studies have indicated ERK1/2 does not show enhanced phosphorylation after swim training in mice [46] or in rats [47], it is clear that ERK1/2 phosphorylation is upregulated in response to pathological hypertrophy stimuli (such as TAC) that induce concentric hypertrophy [43]. Taken together with the inhibited ability of the *Atf6*^*-/-*^ mice to develop hypertrophy and their increased chamber dilation after swim training, it is possible that increased ERK1/2 phosphorylation in *Atf6*^*-/-*^ mice after long-term swim training is indicative of an aberrant response to exercise training. Since ERK1/2 is one of many signals downstream of Ras that participate in a broad growth and protein production response, and loss of ATF6 is known to result in the accumulation of misfolded proteins during hypertrophy [48], we surmise that this interaction may be involved in the reduced cardiac function we observe.

We also found that ACLY phosphorylation at Ser455 was significantly increased in the *Atf6*^-/-^ mice compared to the Wt and *Atf6b*^-/-^ mice (Fig 6). Phosphorylation at Ser455 has been shown to increase the activity of ACLY [49,50], which is an important regulator of cardiac lipid synthesis and contractile function via its roles in metabolism and histone acetylation [51,52]. However, ACLY has been shown to have differential functions in the heart [51–53]. ACLY expression increased after pressure overload [52] but was reduced in human heart failure patients [51,52]. ACLY deletion/inhibition was also associated with reduced cardiac function and chamber dilation [51,52]. ACLY is also required for myofibroblast differentiation and ACLY inhibition has been suggested as a potential target to intervene in the progression of pathological fibrosis [53]. Based on this information, P-ACLY could have been significantly increased in the *Atf6*^-/-^ mice as a compensatory mechanism to maintain cardiac function. Notably, fatty acid synthase, which acts downstream of ACLY in lipid synthesis also showed a strong trend towards upregulation in swim-trained *Atf6*^-/-^ mice that may point to a broader increase in lipid synthesis. However, because of the complex nature of ACLY’s involvement in multiple cell types within the heart during cardiac hypertrophy and the whole-body *Atf6*^-/-^ mouse model used for this study, further investigation is required to understand the specific role of ACLY crosstalk with UPR signaling during exercise.

It is unclear why ATF6β is required for pathological cardiac hypertrophy but not physiological hypertrophy. We previously determined that although transgenic mice with overexpression of activated ATF6α or ATF6β shared a partially overlapping pool of gene targets, each paralog also seemed to specifically regulate a unique pool of genes [8]. Blackwood et. al determined that ATF6α regulates hypertrophy by inducing the mTOR pathway protein Rheb [7]. It is possible that Rheb is one of these genes specifically induced by ATF6α, but not ATF6β, though we did not see a significant change in *Rheb* expression in our qPCR results (Fig 4). In fact, despite the differences in cardiac remodeling in swim-trained *Atf6*^*-/-*^ mice, compared to *Atf6b*^-/-^ and Wt groups, there were no significant changes in the expression of ER stress-associated genes. This may be due to long-term compensation by ATF6β, which is a weaker but longer-lived transcription factor compared to ATF6α [12]. Because we harvested tissue at the end of the swimming protocol, it is possible that we are capturing a time point at which ATF6β-mediated regulation of ER stress-associated genes has “caught up” to the loss of ATF6α. However, a full understanding of the mechanistic differences between ATF6α and ATF6β in the context of physiological cardiac hypertrophy will require additional studies to examine the shorter-term hypertrophy time points.

### Institutional review board statement

The animal study protocol was approved by the Institutional Animal Care and Use Committee of the University of Alabama and in accordance with the National Institutes of Health Guidelines for the care and use of laboratory animals (Protocol # 18-08-1483, last approved on 10-7-2024; and 18-08-1487, last approved on 10-14-2024).

## Supporting information

S1 Fig Trichrome staining performed on deparaffinized heart sections taken from Wt, *Atf6* ^*-/-*^, and Atf6b^-/-^ mice after 20-day swim training. Scale bar is 100 μm.
(TIF)

S2 Fig Uncropped blots from Fig 5A. Molecular weight markers (in kD) shown to the left of the blot.
(TIF)

S3 Fig Uncropped blots from Fig 6A. Molecular weight markers (in kD) shown to the left of the blot.
(TIF)

S4 Fig Uncropped blots from Fig 6C. Molecular weight markers (in kD) shown to the left of the blot.
(TIF)

S1 File Supporting information. Spreadsheet includes the mean, SEM, and data point values for all groups Figs 1–6 .
(XLSX)
