# Supplementary figures and images for: ATF6β is not essential for the development of physiological cardiac hypertrophy
Source: PLoS One. 2025 Apr 7;20(4):e0320178. doi: 10.1371/journal.pone.0320178 (PMC11975135; doi:10.1371/journal.pone.0320178)

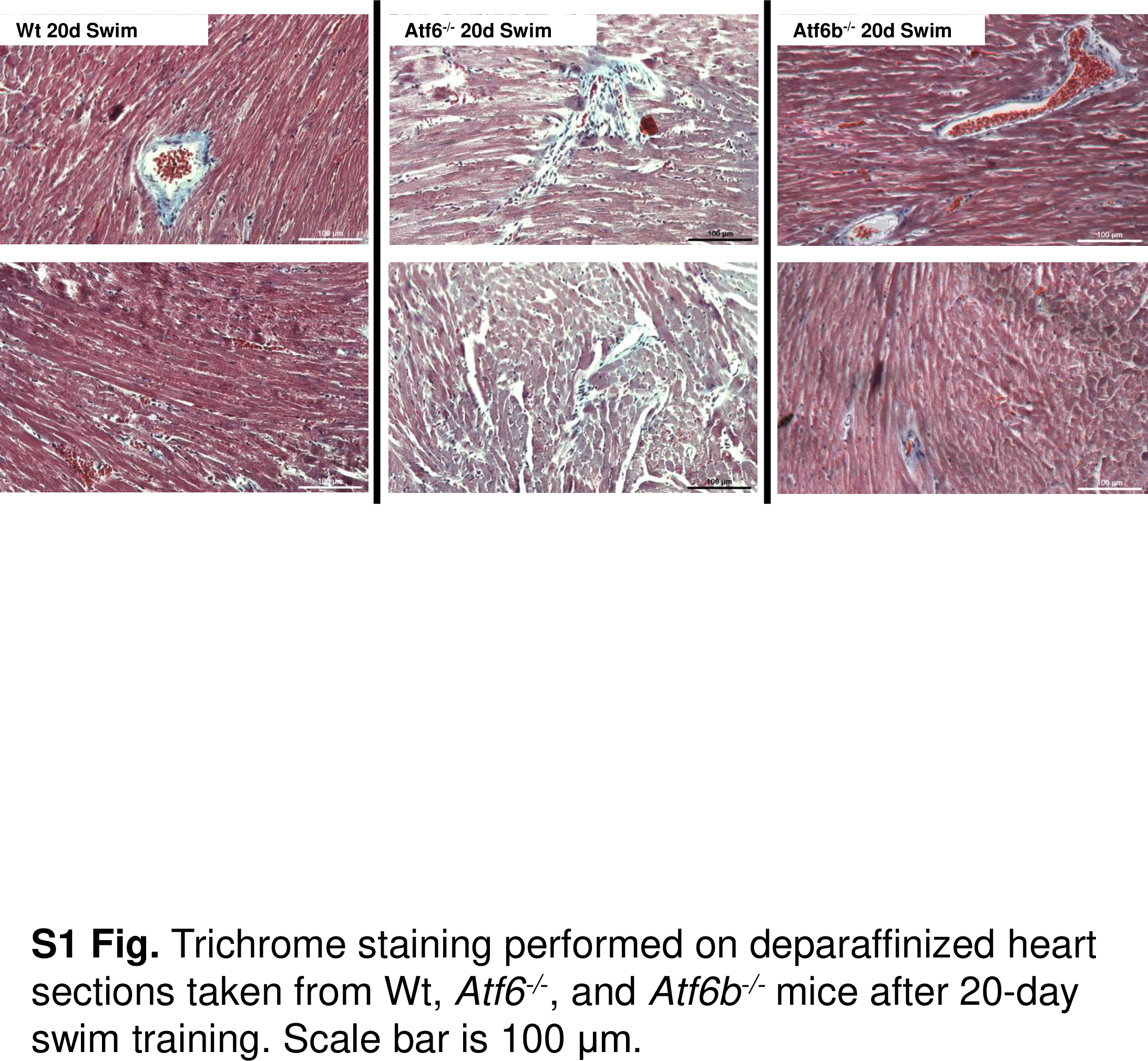

Supplement: S1 Fig — Scale bar is 100 μm. (TIF) [file pone.0320178.s001.tif]

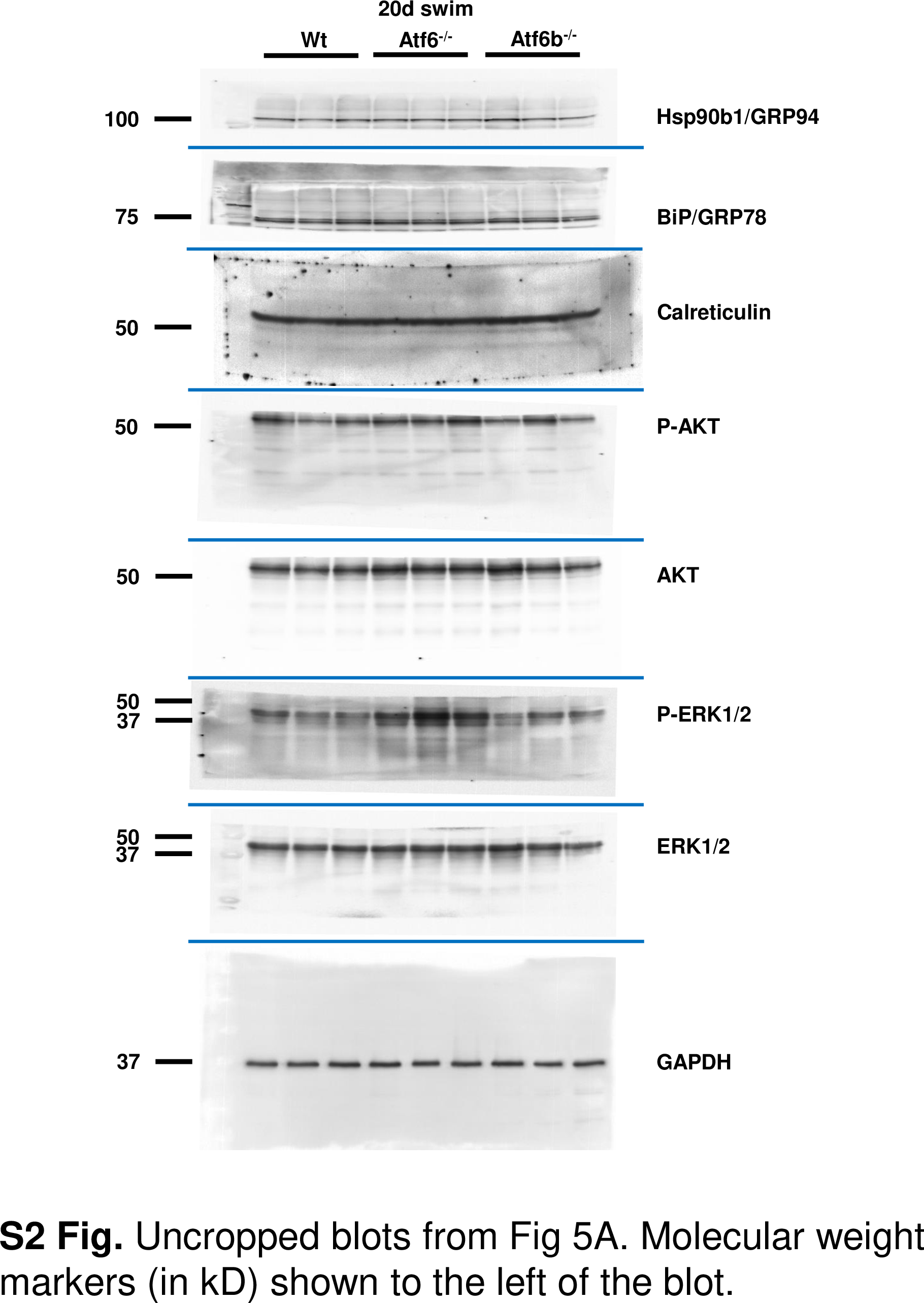

Supplement: S2 Fig — Molecular weight markers (in kD) shown to the left of the blot. (TIF) [file pone.0320178.s002.tif]

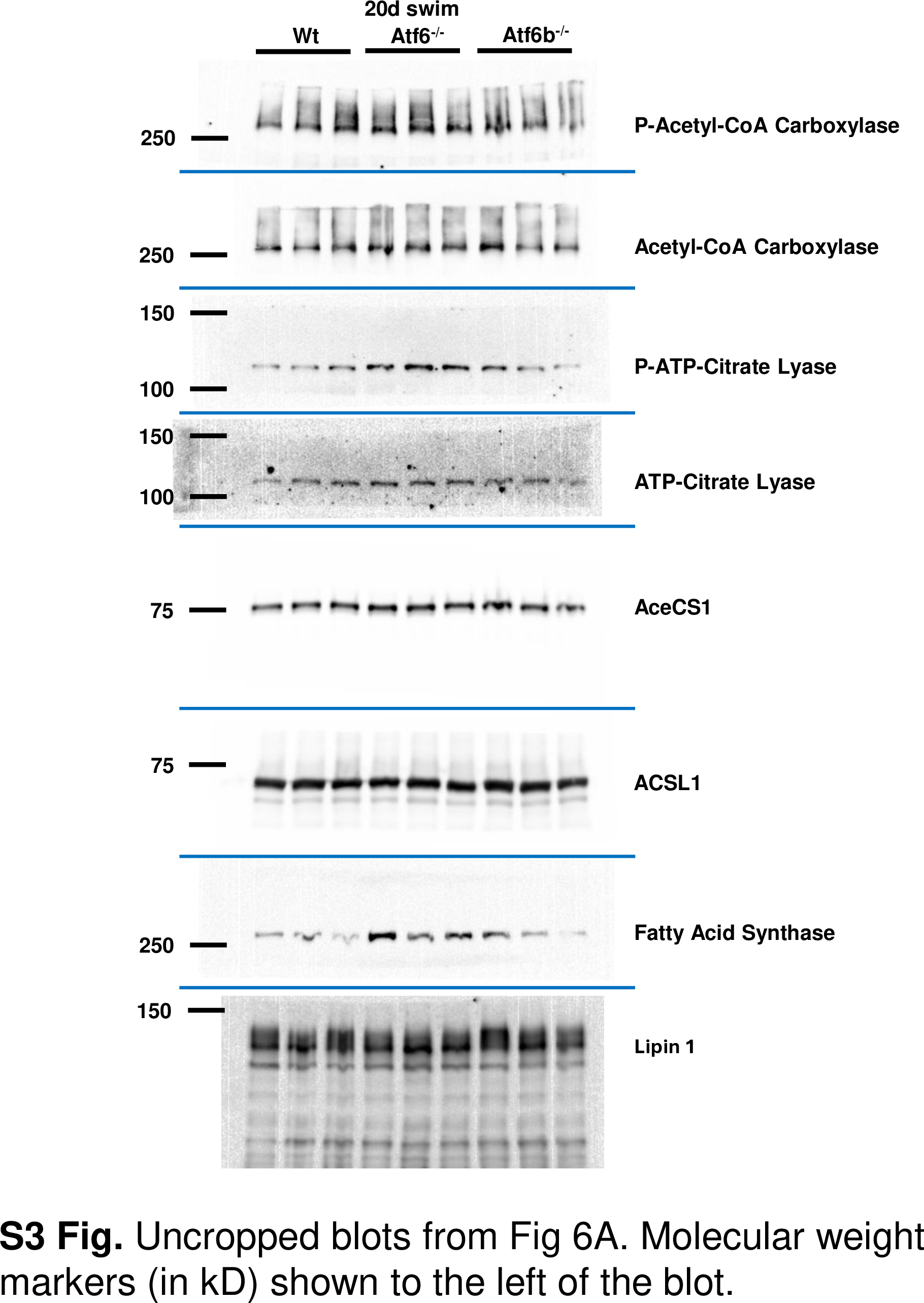

Supplement: S3 Fig — Molecular weight markers (in kD) shown to the left of the blot. (TIF) [file pone.0320178.s003.tif]

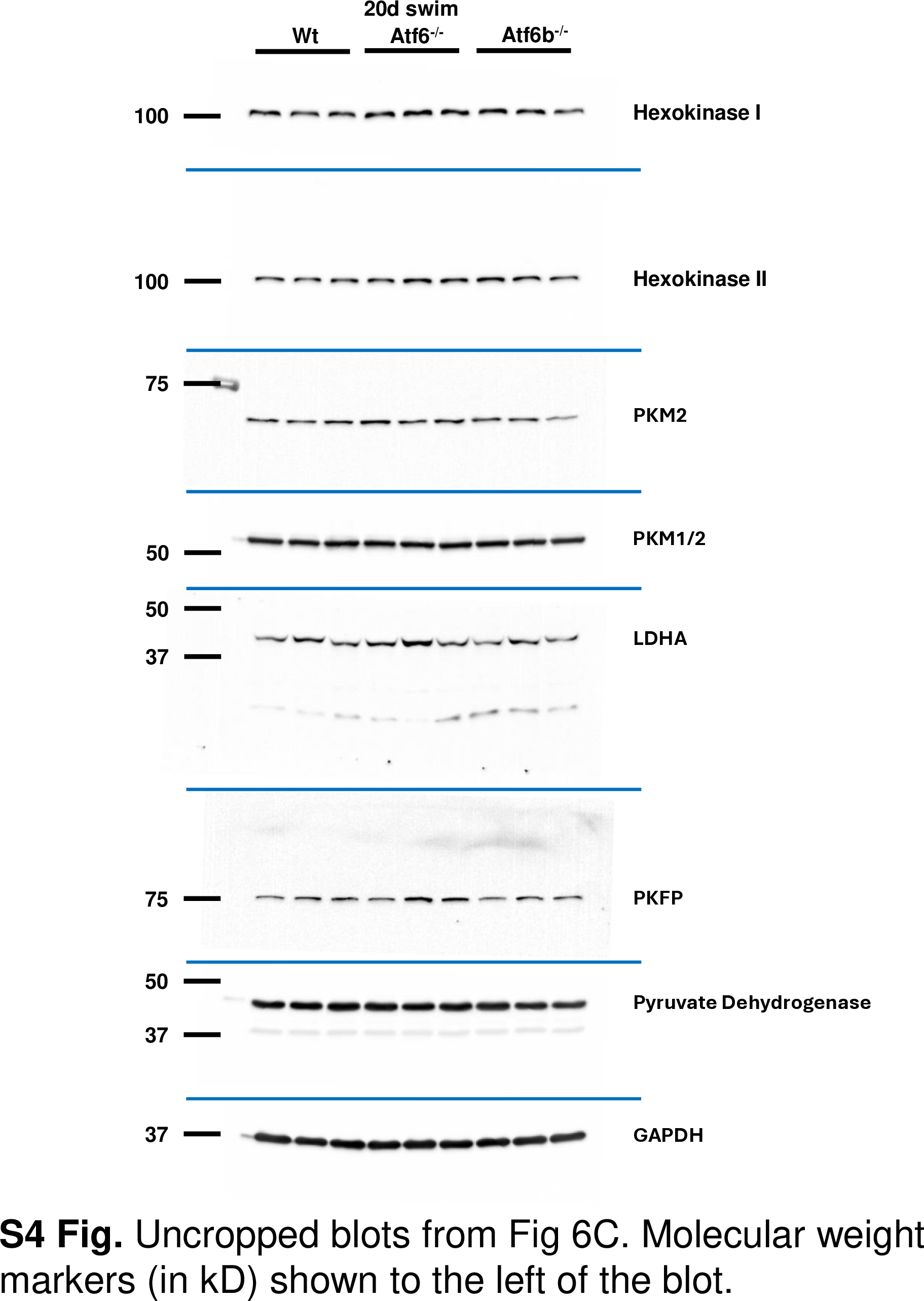

Supplement: S4 Fig — Molecular weight markers (in kD) shown to the left of the blot. (TIF) [file pone.0320178.s004.tif]
